# Combined Contribution of Endothelial Relaxing Autacoides in the Rat Femoral Artery Response to CPCA: An Adenosine A_2_ Receptor Agonist

**DOI:** 10.1100/2012/143818

**Published:** 2012-04-19

**Authors:** Miroslav Radenković, Marko Stojanović, Radmila Janković, Mirko Topalović, Milica Stojiljković

**Affiliations:** ^1^Department of Pharmacology, Clinical Pharmacology and Toxicology, School of Medicine, University of Belgrade, P.O. Box 38, 11129 Belgrade, Serbia; ^2^Institute of Pathology, School of Medicine, University of Belgrade, 11000 Belgrade, Serbia

## Abstract

We examined the contribution of endothelial relaxing factors and potassium channels in actions of CPCA, potent adenosine A_2_ receptor agonist, on isolated intact male rat femoral artery (FA). CPCA produced concentration-dependent relaxation of FA, which was notably, but not completely, reduced after endothelial denudation. DPCPX, A_1_ receptor antagonist, had no significant effect, while SCH 58261 (A_2A_ receptor antagonist) notably reduced CPCA-evoked effect. Pharmacological inhibition of nitric oxide synthase or cyclooxygenase comparably reduced CPCA-evoked action, still in a lesser degree than after denudation. In the presence of buffer with high K^+^ (100 mM), CPCA-produced relaxations were almost abolished. TEA (nonselective K_Ca_ blocker), glibenclamide (K_ATP_ blocker), Ba^++^ (K_IR_ blocker), or ouabain (Na^+^/K^+^-ATPase inhibitor) did not change CPCA-induced relaxation. Concentration-response curve for CPCA was significantly shifted to the right after the incubation of apamin (SK channel blocker). CPCA produced concentration-dependent relaxation of FA that was partly dependent on endothelial cells. Endothelium-related portion of CPCA-elicited effect was mediated by combined action of endothelial NO, prostacyclin, and EDHF after activation of endothelial A_2A_ receptors. Small conductance K_Ca_ channels were involved in this action.

## 1. Introduction

Adenosine is an endogenous purine nucleoside with wide distribution in almost every tissue. It is involved in fine adjustments of numerous physiological functions [1], including vascular tone of different blood vessels [2]. It has been described that local ischemia can induce the release of this nucleoside, which can further modulate vascular resistance and increase oxygen distribution. The vascular effects of adenosine are generally exerted through activation of adenosine receptors, namely, A_1_, A_2A_, A_2B_, and A_3_ receptors, producing contraction or relaxation, this depending on examined blood vessel. Vasodilatations in response to adenosine are predominantly due to activation of adenosine A_2_ receptors located on vascular smooth muscle or endothelial cells [3–8]. Endothelium-independent relaxant effect of adenosine has been demonstrated in guinea pig coronary artery [9], rat inferior mesenteric artery [10], or feline pulmonary artery [11]. On the other hand, adenosine-induced endothelium-dependent relaxations have been recorded in rabbit pulmonary artery [12], rat renal artery [13], or porcine coronary artery [14]. 

Femoral arteries are vital blood vessels allowing blood flow and oxygen supply to the muscles and superficial tissues of legs. However, they are susceptible to the peripheral arterial disease, mainly through atherosclerotic process [15]. Taking into account that actions of adenosine and various agonists of adenosine receptors in femoral arteries of different species are still under investigation, the present experiments were undertaken in order to (1) examine isolated male rat femoral artery response to CPCA—potent A_2_ receptor agonist—[16], (2) evaluate possible contribution of functional endothelium in vascular effects of CPCA, (3) determine contribution of endothelial relaxing factors in CPCA-induced action on femoral artery, and (4) examine if potassium ion channels or Na^+^/K^+^-ATPase is involved in effects of used agonist. 

## 2. Materials and Methods

The standards from the European Convention for the Protection of Vertebrate Animals Used for Experimental and Other Scientific Purposes, as well as guidelines from the Good Laboratory Practice, were fully applied in regard to procedures with used animals. The methodology used in this study was in accordance with our previous investigations [10, 13]. Briefly, the femoral artery was isolated from male Wistar rats (220–280 g), carefully dissected from surrounding fat and connective tissue, cut into circular segments (which were 4 mm long), and immediately placed in the Krebs-Ringer bicarbonate solution (composition in mM: NaCl 118.3, KCl 4.7, CaCl_2_ 2.5, MgSO_4_ 1.2, KH_2_PO_4_ 1.2, NaHCO_3_ 25.0, Ca-EDTA 0.026; glucose 11.1). The endothelium was removed from some rings by gently rubbing the intimal surface with stainless-steel wire. Vascular rings were mounted between two stainless-steel triangles in an organ bath containing, Krebs-Ringer bicarbonate solution (37°C, pH 7.4) and aerated with 95% O_2_ and 5% CO_2_. One of the triangles was attached to a displacement unit allowing a fine adjustment of tension and further connected to a force-displacement transducer (Hugo Sachs Elektronik F30 Type 372, Freiburg, Germany). Isometric tension was continuously recorded on a Rikadenki R-62 multipen electronic recorder (Rikadenki Kogyo CO., Ltd., Tokyo, Japan). 

**Figure 1:**
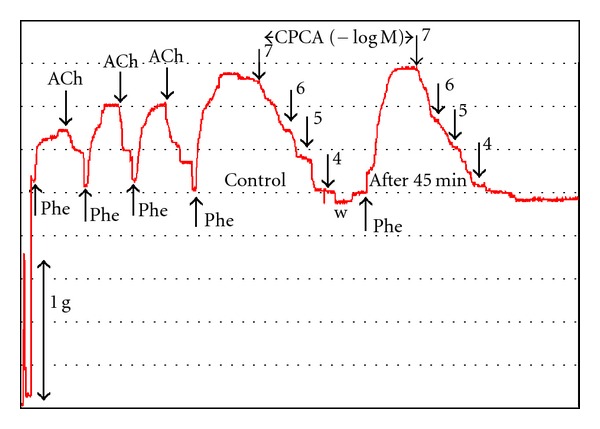
Original recording of CPCA-produced cumulative vascular response of isolated rat femoral artery. A multiple curve design protocol was applied, since the preliminary experiments on preparations of femoral artery (*n* = 4) demonstrated that the first and the second concentration-response curve (determined 45 min apart) for CPCA were not significantly different. Ach: acetylcholine; Phe: phenylephrine.

**Figure 2:**
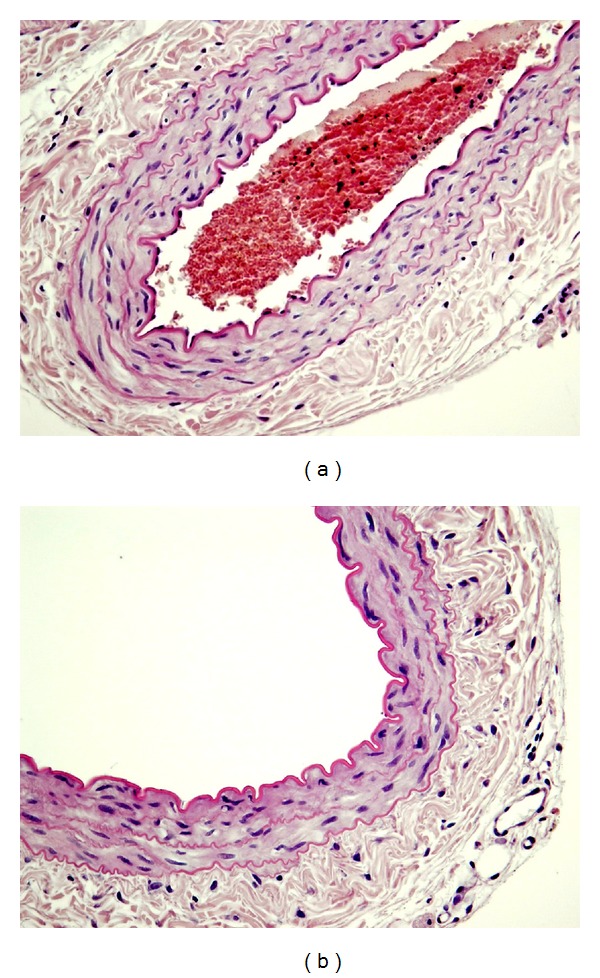
Histological preparations of rat femoral artery with (a), and without (b) endothelial cell layer (hematoxyline and eosin staining, magnification = 400x).

At the beginning of each experiment, endothelium's functional integrity was examined by precontraction of isolated femoral artery with submaximal concentration (EC_50_–EC_70_) of phenylephrine, followed by the addition of acetylcholine (1 *μ*M). This procedure was repeated three times in 20 min intervals (Figure 1). Relaxation >80% of phenylephrine precontraction was indicative for functionally intact endothelium. The morphological integrity of vascular endothelium was additionally confirmed at the end of randomly selected experiments by preparing histological preparations of rat femoral artery with standard haematoxylin and eosin staining (Figure 2). Concentration-response curves for CPCA were obtained in a cumulative fashion on phenylephrine-precontracted arteries. Since separate experiments in preparations of femoral artery demonstrated that the first and the second concentration-response curves (determined 45 min apart) for CPCA were not significantly different, a multiple curve design protocol was applied (Figure 1). Thereafter, all pharmacological blockers were incubated during 30 min prior the second concentration-response curve. 

The effect induced by each concentration of CPCA was expressed as a relaxation percentage of phenylephrine-produced precontraction. The results were expressed as means ± SEM. and *n* refers to the number of experiments. The concentration of CPCA producing 50% of its own maximum response (EC_50_) was determined by using a nonlinear least square fitting procedure of the individual experimental data and was presented as pEC_50_ (pEC_50_ = −log EC_50_). All calculations were done by using the computer program Graph Pad Prism (Graph Pad Software Inc., San Diego, calif, USA). Statistical significance of differences between two means was determined with paired Student's *t*-test. A value of *P* < 0.05 was considered to be statistically significant.

The following chemicals were used: 5′-(N-cyclopropyl) carboxyamidoadenosine (CPCA), indomethacin, 1,3-dipropyl-8-cyclopentylxanthine (DPCPX), 7-(2-phenylethyl)-5-amino-2-(2-furyl)-pyrazolo-[4,3-e]-1,2,4-triazolo[1,5-c] pyrimidine SCH 58261, L-phenylephrine, ouabain, glibenclamide (Sigma, St Louis, USA, Mo); acetylcholine iodide, tetraethylammonium bromide (TEA) (Serva, Heidelberg, Germany); N^G^-nitro-L-arginine (L-NOARG) (RBI, Natick, Mass, USA); apamin, BaCl_2_ (ICN, Irvine, Calif, USA). All agents (except as described below) were dissolved in distilled water and diluted to the desired concentration with buffer. Indomethacin was dissolved in equimolar Na_2_CO_3_ solution, glibenclamide was dissolved in 1,2-propylenglycol, DPCPX was dissolved in DMSO with NaOH, and CPCA stock solution was initially prepared in 0.01 M HCl. Preliminary experiments in preparations of rat femoral artery demonstrated that the vascular action of CPCA was unaffected by used dissolvents. The experiments with ouabain were performed in a dark room. During the experimental procedure, all agents were added directly to the organ bath in a volume of 150 *μ*L, and the concentrations given are the calculated final concentrations in the bath solution.

## 3. Results

**Figure 3:**
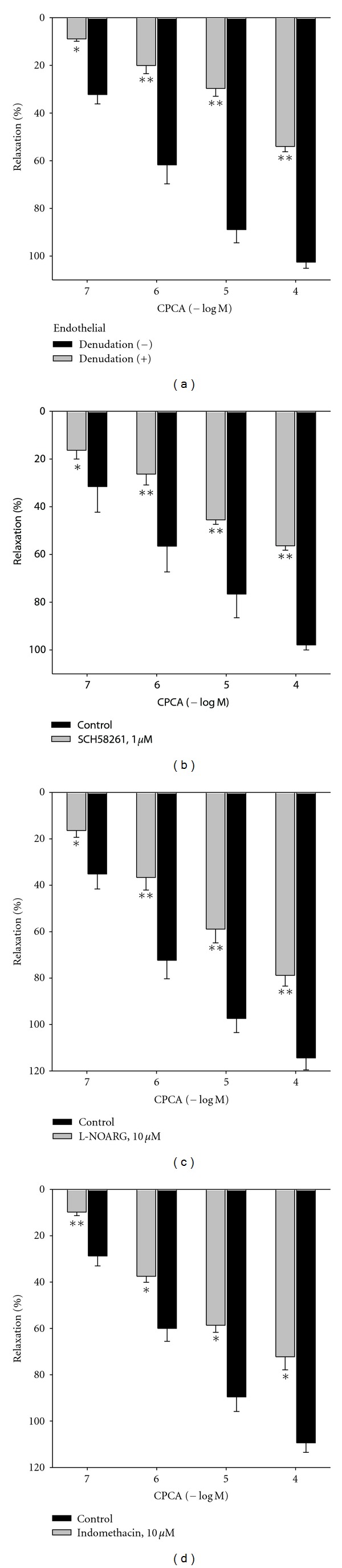
CPCA-induced relaxation in isolated rat femoral artery after the endothelial denudation or in the presence of SCH 58261, N^G^-nitro-L-arginine (L-NOARG), or indomethacin. Each point represents the mean ± SEM. (*n* = 4 − 7). Vascular relaxations induced by CPCA are expressed as percentages of the precontraction induced by phenylephrine. *P < 0.05 and **P < 0.01 compared to the control relaxant effect (*n* = number of vessels).

CPCA (0.1–100 *μ*M) produced concentration-dependent relaxation of intact rat femoral artery. After the endothelial denudation control relaxations induced by CPCA were significantly reduced (*P* < 0.01; Table 1 and Figure 3(a)). Application of DPCPX (10 nM), adenosine A_1_ receptor antagonist, had no significant effect on intact femoral artery relaxation elicited by CPCA (*P* > 0.05; Table 1). On the other hand, SCH 58261 (adenosine A_2A_ receptor antagonist, 1 *μ*M) significantly reduced CPCA-evoked relaxation (*P* < 0.01; Table 1 and Figure 3(b)). Moreover, the maximal relaxant effect of CPCA after the incubation of SCH 58261 was comparable with the one obtained after the endothelial denudation (*P* > 0.05). 

Individual incubations of L-NOARG (NO-synthase inhibitor, 10 *μ*M) and indomethacin (cyclooxygenase inhibitor, 10 *μ*M) produced significant and comparable inhibitions of intact rat femoral artery response to CPCA (Table 1 and Figures 3(c) and 3(d)). Nevertheless, the reduction of maximal relaxant effects, recorded after the incubation of L-NOARG or indomethacin, was less pronounced if compared to the one obtained after the endothelial denudation (Table 1; *P* < 0.05). 

**Figure 4:**
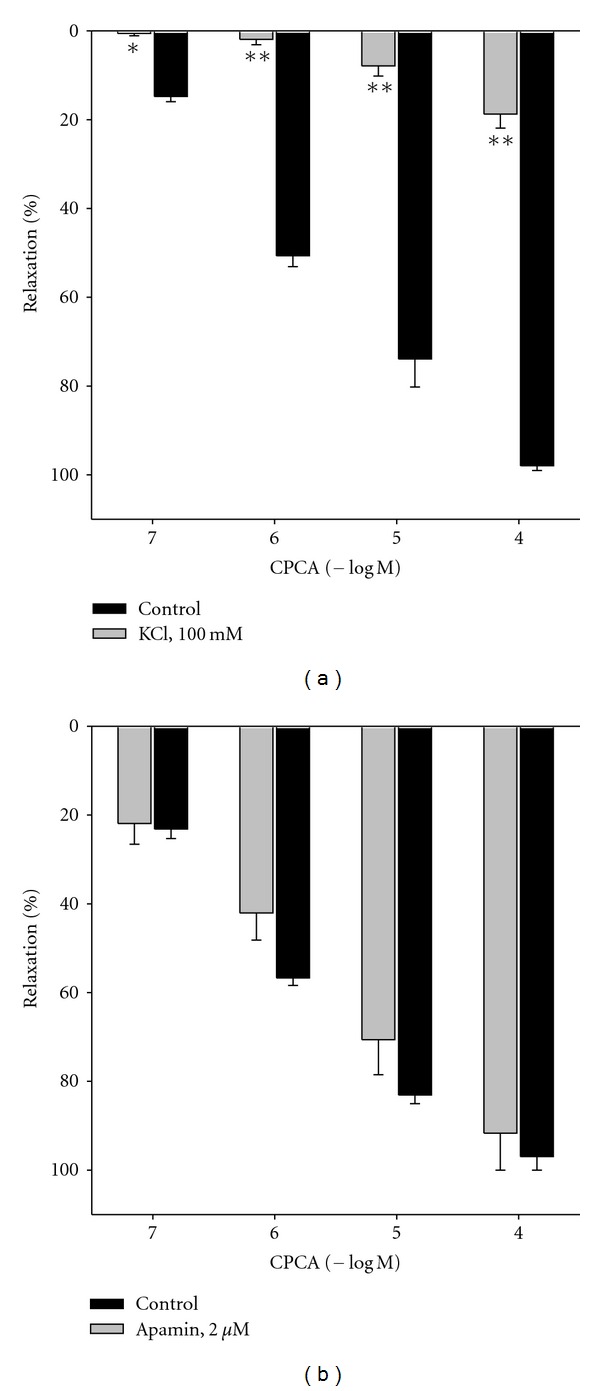
CPCA-induced relaxation in isolated rat femoral artery in the presence of hyperpolarizing solution (KCl = 100 mM) or apamin. Each point represents the mean ± SEM. (*n* = 5 − 7). Vascular relaxations induced by CPCA are expressed as percentages of the precontraction induced by phenylephrine. *P < 0.05 and **P < 0.01 compared to the control relaxant effect (*n* = number of vessels).

In the presence of buffer with high potassium (KCl = 100 mM) CPCA-induced relaxation on intact vascular rings was strongly inhibited (*P* < 0.01; Table 1 and Figure 4(a)). Moreover, control concentration-response curve for CPCA was slightly, but significantly and shifted to the right after the incubation of 2 *μ*M apamin (small conductance K_Ca_ channel blocker; *P* < 0.05; Table 1 and Figure 4(b)). Oppositely, TEA (K^+^ channel blocker applied in a concentration of 500 *μ*M selective for large conductance K_Ca_ channels), glibenclamide (K_ATP_ channel blocker, 1 *μ*M), Ba^++^ (K_IR_ channel blocker, 3 *μ*M), or ouabain (Na^+^/K^+^-ATPase inhibitor, 100 *μ*M) did not change control relaxations induced by CPCA on examined blood vessel (*P* > 0.05; Table 1). 

## 4. Discussion

In our investigation, CPCA induced concentration-dependent relaxant response in intact vascular rings of rat femoral artery. In accordance, bolus injections of adenosine or CPCA into the femoral artery of rabbit produced dose-dependent increase in femoral blood flow and decrease in resistance [17]. After the endothelial denudation, CPCA-produced relaxation in our experiments was notably, yet not completely reduced. Thus, it could be proposed that the effect of CPCA is partly dependent upon intact endothelial cells and feasible release of endothelial relaxing factors. Moreover, it can be also suggested that a significant part of CPCA vascular action is most probably induced by direct action on specific binding sites located on smooth muscle cells.

In order to characterize the subtype of adenosine receptors associated with CPCA action, we used DPCPX and SCH 58261, adenosine A_1_ and A_2A_ receptor antagonists, respectively. As expected, DPCPX did not change control concentration-response curves for CPCA, thus, excluding possible contribution of adenosine A_1_ receptors in femoral artery response to the used agonist. This finding is also supported by the previous result that vasodilatation of the rat femoral artery induced by 2-octynyladenosine (YT-146), an adenosine A_2_ receptor agonist, was not antagonized by DPCPX [18]. On the other hand, in our investigation SCH 58261 significantly reduced CPCA-evoked relaxant effect. Moreover, the maximal relaxant action of CPCA after the incubation of SCH 58261 was statistically comparable with the one obtained after the endothelial denudation. This is indicative for the presumption that the endothelium-dependent part of CPCA-evoked action is most probably initiated by predominant CPCA binding to adenosine A_2A_ receptors located on endothelial cells. Furthermore, it is reasonable to suggest that the endothelium-independent part of the CPCA-produced effect was probably associated with activation of another adenosine A_2_ receptor subtype, most likely A_2B_, which is located on smooth muscle cells. This hypothesis needs further evaluation.

In the next part of our study, it was of interest to evaluate possible contribution of endothelial nitric oxide and prostacyclin in vascular response to CPCA. Thus, CPCA-evoked relaxation was notably reduced by L-NOARG. This was in accordance with partial inhibition of rat femoral artery response to 2-octynyladenosine (YT-146), adenosine A_2_ receptor agonist, obtained in another investigation after the incubation of L-NNA (also an NO-synthase inhibitor) [18]. Moreover, in a different study NO-synthase inhibition induced by L-NAME greatly reduced the increase in femoral vascular conductance evoked by CGS 21680, agonist of adenosine A_2A_receptors [19]. However, in our experiments the reduction recorded after incubation of L-NOARG was less pronounced if compared with the one obtained after endothelial denudation. This suggests that endothelial nitric oxide is only partially involved in endothelium-dependent fraction of the transduction mechanism involved in rat femoral artery response to CPCA. Concurrently, based on additionally performed experiments with indomethacin, the same presumption can be proposed regarding the involvement of endothelial prostacyclin. 

The corresponding analysis of previous results showed that endothelial nitric oxide and prostacyclin equally contributed to CPCA-induced relaxation of investigated blood vessel. Moreover, as mentioned earlier, the obtained reduction in the presence of L-NOARG or indomethacin was significantly lower than that after endothelial denudation. This was indicative for the assumption that separate endothelium-dependent component of CPCA-evoked effect was not completely mediated by nitric oxide or prostacyclin. Thus, we hypothesized that some other endothelial relaxing factor, presumably endothelium-derived hyperpolarizing factor (EDHF), may be involved in CPCA-induced effect. This assumption was confirmed in our experiments in which potassium conductance was blocked by buffer with high K^+^ (100 mM) resulting in strong inhibition of the CPCA-induced relaxation. Thus, it can be proposed that EDHF notably contributed to the endothelium-dependent fraction of CPCA action. However, if we take into account that the inhibition in the presence of high potassium buffer was more pronounced than the one recorded after endothelial denudation, it can be assumed that, apart from the indirect action mediated via EDHF, a significant part of CPCA effect was actually a result of direct action on smooth muscle cells and subsequent increase of cellular membrane potassium conductance. Although this suggestion requires secondary experimental assessment, in our recent investigation on femoral artery we obtained that adenosine-induced relaxation was endothelium-independent and fully inhibited in the presence of a buffer with high K^+^ [20].

In general, EDHF-mediated vascular effects can be exerted through activation of Na^+^/K^+^-ATPase or different potassium channels [21–23]. In our study, TEA (potassium channel blocker applied in a concentration selective for large conductance calcium-activated potassium channels—BK_Ca_), glibenclamide (ATP-sensitive potassium channel blocker—K_ATP_), Ba^++^ (inward-rectifying potassium channel blocker—K_IR_), or ouabain (Na^+^/K^+^-ATPase inhibitor) did not change control relaxations induced by CPCA on examined blood vessel. This excluded the possible role of blocked pharmacological targets in femoral artery relaxant response to CPCA. On the other hand, the control pEC_50_ value for CPCA-produced concentration-response curve was significantly reduced after the incubation of apamin, which suggests certain role of small conductance calcium-activated potassium channels (SK) in studied vasorelaxant process.

In conclusion, CPCA produced concentration-dependent relaxation of isolated rat femoral artery that was partly dependent on endothelial cells. Endothelium-related part of the CPCA-elicited effect was mediated by combined action of endothelial nitric oxide, prostacyclin, and EDHF after predominant activation of endothelial adenosine A_2A_ receptors. Small conductance K_Ca_ channels were involved in transduction mechanism of CPCA-induced vascular response on examined blood vessel. It appears that endothelium-independent portion of CPCA-evoked relaxation of rat femoral artery did not involve adenosine A_2A_ receptors. This direct effect of CPCA on smooth muscle cells was most likely dependent upon activation of adenosine A_2B_ receptors and subsequent increase of cellular membrane potassium conductance. However, these assumptions need further clarification. 

## Figures and Tables

**Table 1:** CPCA-induced relaxation in isolated rat femoral artery in the absence or in the presence of the specific pharmacological intervention. Concentration of CPCA eliciting 50% of its own maximum response (EC_50_) is presented as pEC_50_ (pEC_50_ = −log EC_50_), whereas maximal obtained relaxation is expressed as a percentage of the precontraction produced by phenylephrine. Each result represents the mean ± SEM (*n* = 4 − 7).

Pharmacological intervention	pEC_50_ ± SEM	Max (%) ± SEM	Pharmacological intervention	pEC_50_ ± SEM	Max (%) ± SEM
Denudation (−)	5.91 ± 0.17	102.56 ± 2.56	TEA (−)	6.05 ± 0.18	96.34 ± 0.97
Denudation (+)	4.79 ± 0.38*	54.01 ± 2.22*	TEA (+)	6.30 ± 0.18	85.50 ± 13.30
DPCPX (−)	5.99 ± 0.27	96.66 ± 5.89	Apamin (−)	5.90 ± 0.19	96.97 ± 3.03
DPCPX (+)	5.88 ± 0.23	89.94 ± 7.62	Apamin (+)	5.47 ± 0.25*	91.67 ± 8.33
SCH 58261 (−)	5.71 ± 0.42	97.9 ± 2.09	Glibenclamide (−)	5.84 ± 0.45	101.92 ± 1.92
SCH 58261 (+)	5.46 ± 0.12*	56.35 ± 1.84*	Glibenclamide(+)	5.76 ± 0.26	89.28 ± 6.83
L-NOARG (−)	6.03 ± 0.25	114.42 ± 8.11	Ba^++^ (−)	6.13 ± 0.42	99.98 ± 0.49
L-NOARG (+)	5.51 ± 0.35*	78.81 ± 4.62*	Ba^++^ (+)	6.01 ± 0.38	95.32 ± 3.94
Indomethacin (−)	5.82 ± 0.26	109.37 ± 4.13	Ouabain (−)	6.09 ± 0.26	100.49 ± 1.12
Indomethacin (+)	5.96 ± 0.24	72.25 ± 5.62*	Ouabain (+)	6.12 ± 0.28	104.32 ± 2.18
High K^+^ (−)	5.92 ± 0.39	97.94 ± 1.09			
High K^+^ (+)	4.70 ± 0.04*	18.76 ± 3.13*			

*P < 0.05 compared to the respective control (paired Student's *t*-test).
